# Data supporting the involvement of the adenine nucleotide translocase conformation in opening the Tl^+^-induced permeability transition pore in Ca^2+^-loaded rat liver mitochondria

**DOI:** 10.1016/j.dib.2016.03.030

**Published:** 2016-03-11

**Authors:** Sergey M. Korotkov

**Affiliations:** Sechenov Institute of Evolutionary Physiology and Biochemistry, the Russian Academy of Sciences, Thorez pr. 44, 194223 St. Petersburg, Russian Federation

**Keywords:** Tl^+^, Ca^2+^, Oxygen consumption assay, Mitochondrial swelling, Mitochondrial membrane potential, Rat liver mitochondria

## Abstract

There we made available information about the effects of the adenine nucleotide translocase (ANT) ‘c’ conformation fixers (phenylarsine oxide (PAO), tert-butylhydroperoxide (tBHP), and carboxyatractyloside) as well as thiol reagent (4,4′-diisothiocyanostilbene-2,2′-disulfonate (DIDS)) on isolated rat liver mitochondria. We observed a decrease in A_540_ (mitochondrial swelling) and respiratory control rates (RCR_ADP_ [state 3/state 4] and RCR_DNP_ [2,4-dinitrophenol-uncoupled state/basal state or state 4]), as well as an increase in Ca^2+^-induced safranin fluorescence (F_485/590_, arbitrary units), showed a dissipation in the inner membrane potential (ΔΨ_mito_), in experiments with energized rat liver mitochondria, injected into the buffer containing 25–75 mM TlNO_3_, 125 mM KNO_3_, and 100 µM Ca^2+^. The fixers and DIDS, in comparison to Ca^2+^ alone, greatly increased A_540_ decline and the rate of Ca^2+^-induced ΔΨ_mito_ dissipation. These reagents also markedly decreased RCR_ADP_ and RCR_DNP_. The MPTP inhibitors (ADP, cyclosporin A, bongkrekic acid, and N-ethylmaleimide) fixing the ANT in ‘m’ conformation significantly hindered the above-mentioned effects of the fixers and DIDS. A more complete scientific analysis of these findings may be obtained from the manuscript “To involvement the conformation of the adenine nucleotide translocase in opening the Tl^+^-induced permeability transition pore in Ca^2+^-loaded rat liver mitochondria” (Korotkov et al., 2016 [1]).

**Specifications Table**

**Table:** 

Subject area	Biology
More specific subject area	Biochemical toxicology
Type of data	Table
How data was acquired	Observational data, swelling assay as a decline in A_540_, oxygen consumption assay, mitochondrial membrane potential assay as safranin fluorescence intensity change at 485/590 nm
Data format	Raw and analyzed
Experimental factors	Temperature and concentration of TlNO_3_ in buffers
Experimental features	Liver was extracted from Wistar male (250–300 g). Rat liver mitochondria were isolated by a dual sequential isolation, and the resulting protein was used for the observational data assay
Data source location	St. Petersburg, Russian Federation
Data accessibility	Data is within the article.

**Value of the data**

• The scientific data can be referenced by other scientists investigating the effects of Tl^+^ on cells and mitochondria.
• The findings can provide comprehensive toxicological analysis of the effects of thallous salts on animal organisms.
• Effects of tBHP, PAO, and DIDS in the new in vitro model of the K^+^surrogate Tl^+^-induced MPTP can be the basis in searching new inducers and inhibitors of mitochondrial permeability transition pores in the inner membrane.
• These data may be helpful in evaluating the combined action of thallium and other sulfhydryl toxicants such as heavy metals and industrial oxidants.

## 1 Data

This manuscript contains additional information to the research of [1]. The use of swelling technique as the change in A_540_ tests changes in mitochondrial volume. The respiratory control ratios (RCR_ADP_=state 3/state 4 and RCR_DNP_=DNP-uncoupled respiration/basal state or state 4) give information about enzymes, involved in oxygen consumption and oxidative phosphorylation processes, correspondingly. The safranin uptake of energized rat liver mitochondria allows to do assertion about the change in the inner membrane potential (ΔΨ_mito_).

## 2 Experimental design, materials and methods

The research was used male Wistar rats (250–300 g) of 9–12 months old which were kept at 20–23 °C under 12-h light/dark cycle with free access to water ad libitum and the standard rat diet. All treatment procedures of rats were carried out according to the Animal Welfare act and the Institute Guide for Care and Use of Laboratory Animals.

### 2.1 Isolation of rat liver mitochondria

Rat liver mitochondria were isolated accordance the standard protocol [2]. Male rat was decapitated and the liver was quickly extracted and placed into ice-cold isolation buffer containing 250 mM sucrose, 3 mM Tris–HCl (pH 7.3), and 0.5 mM ethylene glycol tetraacetic acid (EGTA). The decapitation procedure of fasted animals is mandatory in isolating rat liver mitochondria. Then the liver was minced with scissors, washed out by the medium, transferred into a Potter-Elvehjem glass homogenizer and homogenized using a teflon pestle. The liver homogenate was centrifuged at 800×*g* for 7.5 min, then the pellet has been thrown out and the supernatant was centrifuged at 10,000×*g* for 10 min. The mitochondrial pellet was twice washed out with a buffer containing 250 mM sucrose and 3 mM Tris–HCl (pH 7.3) and centrifuged at 10,000×*g* for 10 min. The final pellet was resuspended in 950 μl of the wash buffer and kept on ice during the experiment. The whole process of mitochondrial isolation was carried out on ice. The mitochondrial protein content was determined by Bradford [3] and was within the range of 50–60 mg/ml.

### 2.2 Swelling of mitochondria

The early mention about suitability to use millimolar Tl^+^ concentrations was made in research of Melnick et al. and Saris et al. which applied swelling and polarographic techniques in experiments with isolated mitochondria (see more detail [1]). The applicability of such experimental model in toxicological studies using isolated mitochondria and buffers containing thallous salts has been earlier substantiated by us in more detail [4]. Mitochondrial swelling was measured as a decrease in A_540_ at 20 °C using a SF-46 spectrophotometer (LOMO, St. Petersburg, Russia). Mitochondria (1.5 mg protein/ml) were injected into a 1-cm cuvette with 1.5 ml of 400 mOsm buffer containing 200 mM KNO_3_ (Table 1) or 75 mM TlNO_3_ and 125 mM KNO_3_ (Table 1, Table 2, Table 3, Table 4) as well as 5 mM succinate, 5 mM Tris–NO_3_ (pH 7.3), 2 μM rotenone, and 1 μg/ml of oligomycin. The following chemicals were added into the medium before mitochondria: phenylarsine oxide (PAO), tert-butyl hydroperoxide (tBHP), N-ethylmaleimide (NEM), 4,4′-diisothiocyanostilbene-2, 2′-disulfonate (DIDS), ADP, cyclosporin A (CsA), bongkrekic acid (BKA), and carboxyatractyloside (CATR). Ca^2+^ (where indicated) was injected to the buffer at one min after mitochondria. The swelling, oxygen consumption rates, and ΔΨ_mito_ were carried out in 400 mOsm media in order to verify the comparability and consistency between findings in different experiments.

### 2.3 Oxygen consumption assay

**Fig. 1:**
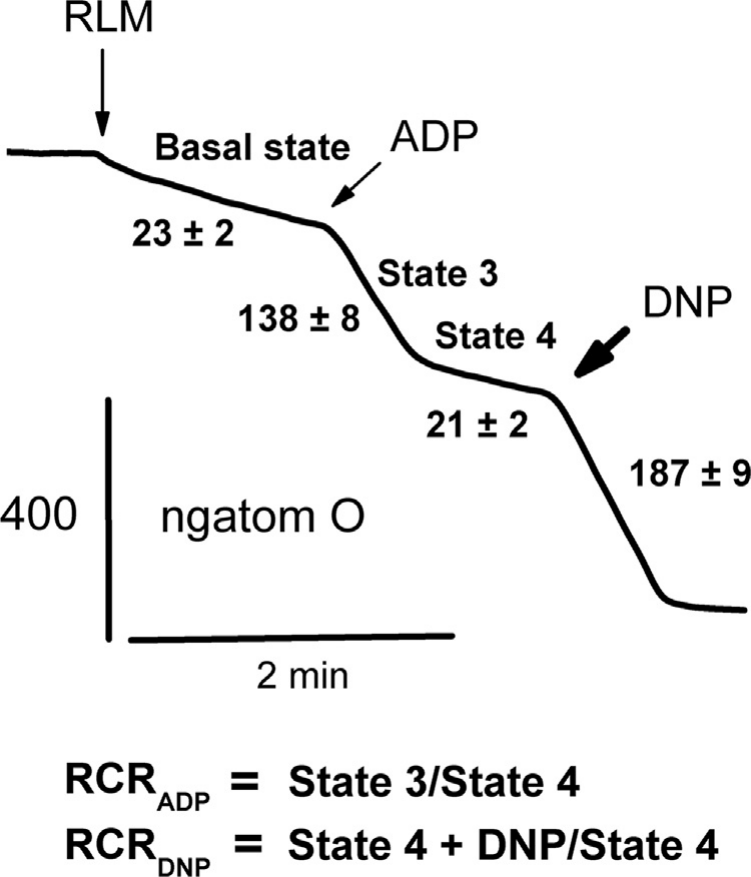
Typical traces in vitro research of rat liver mitochondria. Mitochondria (1.5 mg/ml of protein) were added in medium containing 100 mM KCl, 20 mM Tris–HCl (pH 7.3), 3 mM MgCl_2_, and 3 mM Tris–PO_4_, 5 mM Tris–succinate, and 2 µM rotenone. Additions of mitochondria (RLM), 130 μM ADP (ADP), and 30 μM DNP (DNP) showed by arrows. Oxygen consumption rates (ng atom O min/mg of protein) are presented as numbers placed near experimental traces.

Respiration (oxygen consumption rate) was tested using Expert-001 analyzer (Econix-Expert Ltd., Moscow, Russia) in a 1.3-ml closed thermostatic chamber with magnetic stirring at 26 °C. Mitochondria (1.5 mg protein/ml) were administrated into 400 mOsm buffer containing 25 mM TlNO_3_, 100 mM sucrose, 3 mM Mg(NO_3_)_2_, and 3 mM Tris-PO_4_ (Table 5) or 75 mM TlNO_3_ and 1 μg/ml of oligomycin (Table 6) as well as 125 mM KNO_3_, 5 mM Tris–NO_3_ (pH 7.3), 5 mM succinate, and 2 μM rotenone. In some cases, we used buffers containing glutamate with malate and free of rotenone (Fig. 1). The following reagents (Table 5) were added in the buffer at one minute after mitochondria: PAO, tBHP, DIDS, and NEM. ADP at 130 μM and DNP at 30 μM were correspondingly injected into the buffer after 2 min to induce state 3 and after 4 min to record DNP-uncoupled respiration. The following reagents (Table 6) were added in the buffer one min after mitochondria: PAO, tBHP, and DIDS. If the MPTP inhibitors (ADP plus CsA or NEM alone) were injected into the buffer one min after mitochondria, the first reagents (PAO, tBHP, and DIDS) and Ca^2+^ at 100 μM were correspondingly added into the buffer one and two min latter the inhibitors. Further, DNP at 30 μM was administrated one minute later the reagents or Ca^2+^ (Table 6). The respiratory control ratio (RCR_ADP_=state 3/state 4) that shows the quality of rat liver mitochondria (RLM) was measured in standard buffer containing 100 mM KCl, 20 mM Tris–HCl (pH 7.3), 3 mM MgCl_2_, and 3 mM Tris–PO_4_, 5 mM Tris–succinate, and 2 µM rotenone. The RCR_ADP_ for succinate-energized RLM was equal 6.65±0.21 (*n*=14) (Fig. 1). Wherein, the DNP-dependent respiratory control ratio (RCR_DNP_) was calculated as a ratio of DNP-uncoupled respiration to state 4 (Fig. 1). The RCR_DNP_ for succinate-energized RLM was equal 9.18±0.49 (*n*=14) (Fig. 1). Table 5 shows the RCR_ADP_ under above experimental conditions in TlNO_3_ buffers. The DNP-dependent respiratory control ratio (RCR_DNP_) in above TlNO_3_ buffers was accordingly determined as a ratio of DNP-uncoupled respiration to state 4 (Table 5) or a basal state respiration (Table 6).

### 2.4 Mitochondrial membrane potential

The ΔΨ_mito_ induced in succinate-energized on the IMM of RLM (Table 7) was evaluated according to Waldmeier [5] by the intensity of safranin fluorescence (arbitrary units) in the mitochondrial suspension with magnetic stirring at 20 °C using a Shimadzu RF-1501 spectrofluorimeter (Shimadzu, Japan) at 485/590 nm wavelength (excitation/emission). Mitochondria (0.5 mg protein/ml) were placed into a quartz cuvette of four clear walls with 3 ml of a buffer containing 20 mM TlNO_3_, 125 mM KNO_3_, 110 mM sucrose, 5 mM Tris–NO_3_ (pH 7.3), 1 mM Tris-P_i_, 3 μM safranin, 2 μM rotenone, and 1 μg/ml of oligomycin. In addition, the next chemicals were added in the medium before mitochondria: PAO, tBHP, DIDS, ADP, and CsA (where indicated). Succinate, Ca^2+^, and DNP were administrated into the medium after mitochondria. Temperature conditions used in the research were standard for experiments with isolated mitochondria *in vitro*.

### 2.5 Statistics

The statistical differences and *P*-values of experimental results in Table 1, Table 2, Table 3, Table 4, Table 5, Table 6, Table 7 are correspondingly evaluated using the two population *t*-test (Microcal Origin, Version 6.0, Microcal Software).

### 2.6 Chemicals

CaCl_2_, Mg(NO_3_)_2_, H_3_PO_4_, KNO_3_, TlNO_3_, and DNP were of analytical grade from Nevareactiv (St. Petersburg, Russia). Rotenone, oligomycin, PAO, tBHP, NEM, tris–OH, EGTA, ADP, CsA, BKA, CATR, and succinate were from Sigma (St. Louis, MO, USA). DIDS was purchased from Santa Cruz Biotechnology (USA). Sucrose as 1 M solution was refined from cation traces on a column filled with a KU-2-8 resin from Azot (Kemerovo, Russia).

## Figures and Tables

**Table 1:** Effects of PAO and tBHP on A_540_ in suspension of succinate-energized rat liver mitochondria in nitrate buffers.

**PAO** (µM) ↓	**200** **mM KNO**_**3**_		**75** **mM TlNO**_**3**_**+125** **mM KNO**_**3**_		**tBHP** (µM) ↓	**200** **mM KNO**_**3**_		**75** **mM TlNO**_**3**_**+125** **mM KNO**_**3**_	
	ΔA_540_±SEM	*P* value	ΔA_540_±SEM	*P* value		ΔA_540_±SEM	*P* value	ΔA_540_±SEM	*P* value
0	−0.030±0.003 (3)	–	−0.027±0.001 (3)	–	0	−0.031±0.002 (3)	–	−0.040±0.009 (3)	–
1			−0.094±0.012 (3)	*P*<0.05	50			−0.100±0.006 (3)	*P*<0.02
2			−0.248±0.022 (3)	*P*<0.01	75			−0.218±0.015 (3)	*P*<0.01
5	−0.092±0.018 (3)	*P*<0.03	−0.534±0.011 (3)	*P*<0.01	100			−0.412±0.006 (3)	*P*<0.01
10	−0.447±0.040 (3)	*P*<0.01	−0.656±0.025 (3)	*P*<0.01	200			−0.415±0.007 (3)	*P*<0.01
20	−0.632±0.019 (3)	*P*<0.01	−0.715±0.023 (3)	*P*<0.01	500	−0.049±0.005 (3)	*P*<0.03	−0.418±0.007 (3)	*P*<0.01


The absorbance changes (ΔA_540_) were detected within seven minute interval after addition of mitochondria and presented as Means±SEM. The number of experiments showed in parentheses. *P*-values were accordingly calculated to experiments free of PAO or tBHP (a dash in the *P* value columns).

**Table 2:** Effects of PAO, DIDS, and tBHP on change of A_540_ in suspension of Ca^2+^-loaded succinate-energized rat liver mitochondria.

**PAO** (µM) ↓	**−ADP**		**+500** **µM ADP**		**tBHP** (µM) ↓	**−ADP**		**+500** **µM ADP**	
	ΔA_540_±SEM	*P* value	ΔA_540_±SEM	*P* value		ΔA_540_±SEM	*P* value	ΔA_540_±SEM	*P* value
0	−0.197±0.006 (8)	–	−0.013±0.001 (8)	*P*<0.01	0	−0.203±0.010 (9)	–	−0.015±0.002 (9)	*P*<0.01
1	−0.241±0.014 (3)	*P*<0.01	−0.025±0.002 (3)	*P*<0.01	25	−0.213±0.015 (3)	*	−0.017±0.003 (3)	*P*<0.01
2	−0.255±0.006 (6)	*P*<0.01	−0.078±0.007 (8)	*P*<0.01	50	−0.219±0.009 (8)	*	−0.143±0.027 (7)	*P*<0.04
5	−0.305±0.018 (3)	*P*<0.01	−0.279±0.015 (3)	*	100	−0.235±0.010 (4)	*	−0.248±0.011 (4)	*
10	−0.363±0.013 (3)	*P*<0.01	−0.349±0.016 (3)	*	500	−0.230±0.017 (3)	*	−0.249±0.015 (3)	*
									
									
**DIDS** (µM) ↓	**−Ca**^**2+**^				**DIDS** (µM) ↓	**+100** **µM Ca**^**2+**^			
	**−ADP**		**+500** **µM ADP**			**−ADP**		**+500** **µM ADP**	

	ΔA_540_±SEM	*P* value	ΔA_540_±SEM	*P* value		ΔA_540_±SEM	*P* value	ΔA_540_±SEM	*P* value
0	−0.021±0.002 (3)	─	−0.016±0.001 (3)	*	0	−0.288±0.007 (3)	─	−0.016±0.001 (3)	*P*<0.01
2.5	−0.021±0.001 (3)	*	−0.022±0.001 (3)	*	2.5	−0.303±0.010 (3)	*	−0.016±0.002 (3)	*P*<0.01
5	−0.031±0.001 (3)	*P*<0.03	−0.020±0.001 (3)	*	5	−0.314±0.016 (3)	*	−0.132±0.007 (3)	*P*<0.01
12.5	−0.044±0.001 (3)	*P*<0.02	−0.032±0.001 (3)	*P*<0.03	12.5	−0.376±0.012 (3)	*P*<0.04	−0.272±0.018 (3)	*
25			−0.064±0.002 (3)	*P*<0.01					
50	−0.273±0.012 (3)	*P*<0.01	−0.126±0.006 (3)	*P*<0.01					
100	−0.332±0.018 (3)	*P*<0.01	−0.318±0.018 (3)	*P*<0.01					

The absorbance changes (ΔA_540_) were accordingly detected within three minute interval after administration of mitochondria (“−Ca^2+^” columns) or 100 µM Ca^2+^ to mitochondria (“+100 µM Ca^2+^” columns) and this is presented as Means±SEM. The number of experiments showed in parentheses. *P*-values in experiments free of Ca^2+^(“−Ca^2+^” columns) are calculated to experiments free additions (a dash in the *P* value columns). *P*-values with Ca^2+^-loaded mitochondria (“+100 µM Ca^2+^” columns) are calculated to experiments with Ca^2+^ alone (a dash in the *P* value columns). Asterisks indicate that statistical difference between appropriate ΔA_540_ values is not statistically significant.

**Table 3:** Effects of PAO, DIDS, and tBHP on A_540_ in suspension of Ca^2+^-loaded succinate-energized rat liver mitochondria in the presence of ADP, CsA, and NEM.

**Before mitochondrial additions of MPTP inhibitors**	**2** **µM PAO**		**50** **µM tBHP**		**5** **µM DIDS**	
	ΔA_540_±SEM	*P* value	ΔA_540_±SEM	*P* value	ΔA_540_±SEM	*P* value
Free additions	−0.259±0.007 (7)	–	−0.229±0.011 (8)	-	−0.314±0.016 (3)	–
ADP	−0.073±0.007 (9)	*P*<0.01	−0.086±0.029 (5)	*P*<0.01	−0.132±0.007 (3)	*P*<0.01
CsA	−0.248±0.011 (3)	*	−0.220±0.034 (3)	*	−0.276±0.018 (3)	*
NEM	−0.157±0.030 (5)	*P*<0.03	−0.119±0.027 (3)	*P*<0.01	−0.235±0.039 (3)	*
ADP+NEM	−0.028±0.003 (5)	*P*<0.01	−0.023±0.006 (3)	*P*<0.01	−0.065±0.028 (3)	*P*<0.02
CsA+NEM	−0.070±0.015 (4)	*P*<0.01	−0.111±0.016 (3)	*P*<0.01	−0.122±0.008 (3)	*P*<0.01
ADP+CsA	−0.032±0.006 (3)	*P*<0.01	−0.019±0.005 (3)	*P*<0.01	−0.015±0.003 (3)	*P*<0.01
ADP+CsA+NEM	−0.013±0.002 (3)	*P*<0.01	−0.013±0.001 (3)	*P*<0.01	−0.046±0.011 (4)	*P*<0.01

The absorbance changes (ΔA_540_) were detected within three minute interval after administration of 100 µM Ca^2+^ to mitochondria and presented as Means±SEM. The number of experiments showed in parentheses and corresponding *P*-values calculated to experiments free of above additions (a dash in the *P* value columns). Asterisks indicate that statistical difference between appropriate ΔA_540_ values is not statistically significant.

**Table 4:** Effect of NEM on A_540_ in suspension of Ca^2+^-loaded succinate-energized rat liver mitochondria.

**NEM** (µM)	**−Ca**^**2+**^		**+100** **µM Ca**^**2+**^			
	**−ADP**		**−ADP**		**+500** **µM ADP**	
	ΔA_540_±SEM	*P* value	ΔA_540_±SEM	*P* value	ΔA_540_±SEM	*P* value
0	−0.009±0.001 (3)	–	−0.192±0.008 (4)	–	−0.014±0.002 (4)	*P*<0.01
50	−0.093±0.007 (3)	*P*<0.01	−0.095±0.009 (3)	*P*<0.01	−0.021±0.004 (3)	*P*<0.01
250			−0.206±0.006 (3)	*	−0.220±0.008 (3)	*
500	−0.298±0.009 (3)	*P*<0.01	−0.288±0.007 (4)	*P*<0.01	−0.265±0.023 (4)	P<0.03
						
						
**Additions of reagents**	**+100** **µM Ca**^**2+**^					
	2*	2*				

	ΔA_540_±SEM	*P* value				
None (control)	−0.196±0.008 (5)	–*				
ADP	−0.025±0.003 (5)	P<0.01				
CATR	−0.204±0.005 (4)	*				
CsA	−0.215±0.015 (4)	*				
ADP+CATR	−0.136±0.026 (4)	P<0.05				
BKA	−0.034±0.006 (3)	P<0.01				

The absorbance changes (ΔA_540_) in experiments free of Ca^2+^(“−Ca^2+^” columns) were detected within six minute interval and *P*-values are calculated to experiments free of NEM (a dash in the *P* value columns). The absorbance changes with CaRLM (“+100 µM Ca^2+^” columns) were detected within three minute after administration of Ca^2+^ to mitochondria and they are presented as Means±SEM. *P*-values with Ca^2+^-loaded mitochondria are calculated to experiments with Ca^2+^ alone (a dash in the *P* value columns). Asterisks indicate that statistical difference between appropriate ΔA_540_ values is not statistically significant. The absorbance changes in experiments free of NEM (2*) were detected within six minute after Ca^2+^ administration to mitochondria. *P*-values (2*) are calculated to experiments free of additions (a dash with asterisk in the *P* value columns).

**Table 5:** Effects of PAO, DIDS, tBHP, and NEM on RCR_ADP_ and RCR_DNP_ in energized rat liver mitochondria.

**PAO** (µM)	RCR_ADP_±SEM	*P* value	RCR_DNP_±SEM	*P* value	**tBHP** (µM)	RCR_ADP_±SEM	*P* value	RCR_DNP_±SEM	*P* value
0	2.57±0.11 (3)	–	3.92±0.27 (3)	–	0	2.47±0.09 (3)	–	3.73±0.16 (3)	–
1	2.22±0.06 (3)	*P*<0.05	3.77±0.29 (3)	*	50	2.47±0.10 (3)	*	3.88±0.17 (3)	*
2	2.19±0.05 (3)	*P*<0.04	3.57±0.29 (3)	*	100	2.32±0.06 (3)	*	3.16±0.22 (3)	*P*<0.04
5	1.83±0.08 (3)	*P*<0.05	2.67±0.30 (3)	*P*<0.04	200	2.40±0.06 (3)	*	2.94±0.12 (3)	*P*<0.02
10	1.42±0.14 (3)	*P*<0.03	1.01±0.34 (3)	*P*<0.03					
**DIDS** (µM)	RCR_ADP_±SEM	*P* value	RCR_DNP_±SEM	*P* value	**NEM** (µM)	RCR_ADP_±SEM	*P* value	RCR_DNP_±SEM	*P* value

0	2.31±0.09 (3)	–	4.18±0.13 (3)	─	0	2.47±0.09 (3)	–	3.73±0.16 (3)	–
12.5	1.73±0.23 (3)	*	3.08±0.36 (3)	*P*<0.05	50	2.16±0.01 (3)	*P*<0.03	3.45±0.10 (3)	*
25	1.27±0.12 (3)	*P*<0.02	2.20±0.25 (3)	*P*<0.02	100	1.94±0.04 (3)	*P*<0.01	3.28±0.11 (3)	*
50	1.00 (3)	*P*<0.01	1.73±0.21 (3)	*P*<0.01	200	1.93±0.05 (3)	*P*<0.01	3.14±0.02 (3)	*P*<0.02
100	1.00 (3)	*P*<0.01	1.06±0.01 (3)	*P*<0.01					
0**	1.88±0.13 (3)	─	2.34±0.12 (3)	─					
12.5**	1.57±0.16 (3)	*	2.20±0.31 (3)	*
25**	1.20±0.04 (3)	*P*<0.01	2.19±0.13 (3)	*
50**	1.09±0.03 (3)	*P*<0.01	1.96±0.22 (3)	*
100**	1.02±0.02 (3)	*P*<0.01	2.05±0.07 (3)	*

Values of RCR_ADP_ and RCR_DNP_ in succinate energized mitochondria are presented as Means±SEM. The number of experiments showed in parentheses. *P*-values are calculated to experiments ftee additions of PAO, DIDS, tBHP, or NEM. Asterisks indicate that difference between appropriate values is not statistically significant. Concentrations of DIDS for mitochondria energized by glutamate and malate are marked by two asterisks.

**Table 6:** Effects of PAO, DIDS, and tBHP on RCR_DNP_ in succinate-energized and Ca2+-loaded rat liver mitochondria.

**PAO** (µM)	RCR_DNP_±SEM	*P* value	**DIDS** (µM)	RCR_DNP_±SEM	*P* value	**tBHP** (µM)	RCR_DNP_±SEM	*P* value
−Ca^2+^	2.48±0.03 (3)	*P*<0.01	−Ca^2+^	2.17±0.05 (3)	*P*<0.01	− Ca^2+^	2.48±0.03 (3)	*P*<0.01
+Ca^2+^	0.53±0.06 (3)	–	+Ca^2+^	0.68±0.06 (3)	–	+Ca^2+^	0.53±0.06 (3)	–
1	0.42±0.03 (3)	*	2.5	1.05±0.02 (3)	*P*<0.05	50	0.39±0.03 (3)	*
2	0.38±0.04 (3)	*	5	0.74±0.04 (3)	*	100	0.38±0.05 (3)	*
5	0.35±0.02 (3)	*P*<0.04	12.5	0.87±0.13 (3)	*	50+ADP+CsA	1.43±0.04 (3)	*P*<0.01
2+ADP+CsA	1.81±0.02 (3)	*P*<0.01	5+ADP+CsA	1.47±0.18 (3)	*P*<0.02	50+NEM	1.78±0.09 (3)	*P*<0.01
2+NEM	1.54±0.09 (3)	*P*<0.01	12.5+ADP+CsA	1.69±0.29 (3)	*P*<0.03			
			2.5+NEM	1.45±0.20 (3)	*P*<0.02			
			5+NEM	0.93±0.03 (3)	*P*<0.03			

Values of RCR_DNP_ are presented as Means±SEM. The number of experiments showed in parentheses. *P*-values are calculated to experiments with Ca^2+^ but free additions of PAO, DIDS, tBHP, or NEM. Asterisks indicate that difference between appropriate values is not statistically significant.

**Table 7:** Effects of PAO, DIDS, and tBHP on rates of Ca^2+^-induced ΔΨ_mito_ dissipation (arbitrary unites per min) in succinate-energized rat liver mitochondria in presence of ADP and CsA.

**Additions**	ΔΨ_mito_ dissipation±SEM (3)	*P* value
−Ca^2+^	7±2 (5)	*P*<0.01
+Ca^2+^(alone)	186±13 (5)	–
Ca^2+^+ADP+CsA	4±1 (5)	*P*<0.01
1 µM PAO	628±76 (3)	*P*<0.01
1 µM PAO+ADP+CsA	3±1 (3)	*P*<0.01
50 µM tBHP	603±42 (3)	*P*<0.01
50 µM tBHP+ADP+CsA	7±3 (3)	*P*<0.01
2.5 µM DIDS	102±5 (3)	*P*<0.01
2.5 µM DIDS+ADP+CsA	4±1 (3)	*P*<0.01

Rates of the Ca^2+^-induced dissipation of ΔΨ_mito_ were detected on segments with the maximal drop of the potential and they are presented as Means±SEM. The number of experiments showed in parentheses and corresponding *P*-values calculated to experiments with Ca^2+^ alone.

## References

[1] Korotkov S.M., Konovalova S.A., Brailovskaya I.V., Saris N.E.L. (2016). To involvement the conformation of the adenine nucleotide translocase in opening the Tl^+^-induced permeability transition pore in Ca^2+^-loaded rat liver mitochondria. Toxicol. in vitro.

[2] Korotkov S.M., Brailovskaya I.V., Shumakov A.R., Emelyanova L.V. (2015). Closure of mitochondrial potassium channels favors opening of the Tl^+^-induced permeability transition pore in Ca^2+^-loaded rat liver mitochondria. J. Bioenerg. Biomembr..

[3] Bradford M.M. (1976). A rapid and sensitive method for the quantitation of microgram quantities of protein utilizing the principle of protein-dye binding. Anal. Biochem..

[4] Korotkov S.M., Emelyanova L.V., Konovalova S.A., Brailovskaya I.V. (2015). Tl^+^ induces the permeability transition pore in Ca^2+^-loaded rat liver mitochondria energized by glutamate and malate. Toxicol. in vitro.

[5] Waldmeier P.C., Feldtrauer J.J., Qian T., Lemasters J. (2002). Inhibition of the mitochondrial permeability transition by the nonimmunosuppressive cyclosporin derivative NIM811. Mol. Pharmacol..

