# Supplementary material for: Data supporting the involvement of the adenine nucleotide translocase conformation in opening the Tl+-induced permeability transition pore in Ca2+-loaded rat liver mitochondria
Source: Data Brief. 2016 Mar 11;7:620–9. doi: 10.1016/j.dib.2016.03.030 (PMC4802814; doi:10.1016/j.dib.2016.03.030)
Supplement: Supplementary file 2 — Supplementary material [file mmc2.doc]

Hao-Ran Wang, Phd

Editor-in-Chief

Data in Brief

dib@mgreditorial.com

Dear professor Wang,

I send you my revised manuscript entitled “Data supporting the involvement of the adenine nucleotide translocase conformation in opening the Tl^+^-induced permeability transition pore in Ca^2+^-loaded rat liver mitochondria” by Sergey M. Korotkov. This revised manuscript was improved according to the remarks of the reviewer.

Sincerely,

Dr. Sergey M. Korotkov, PhD in Biochemistry and MS in Chemistry

March 2, 2016

St. Petersburg

Russian Federation
